# Supplementary material for: Meta-Analysis of Mutations in ALOX12B or ALOXE3 Identified in a Large Cohort of 224 Patients
Source: Genes (Basel). 2021 Jan 9;12(1):80. doi: 10.3390/genes12010080 (PMC7826849; doi:10.3390/genes12010080)
Supplement: Supplementary file 1 [file genes-12-00080-s001.pdf]

Table S1: Identified mutations in *ALOX12B* in patients with ARCI

Patient	Year of birth	Sex	Origin	Phenotype	Nucleotid change	Consequences of mutation (amino acid level)
1[a]	1954	f	France	CB	**c.864del / c.1655-7C>A**	**p.(Val289Serfs*63) / p.?**
2[a]	1985	m	France	CB	**c.2021_2022dup / c.1655-7C>A**	**p.(Asp675Thrfs*21) / p.?**
3	1991	m	France	CB	**c.1309A>T** / c.1642C>T	**p.(Ile437Phe)** / p.(Arg548Trp)
4	1988	f	France	CB	c.1272dup / **c.2000A>C**	p.(Lys425Glnfs*24) / **p.(Gln667Pro)**
5	1983	f	France	CB	c.1654+3A>G / c.1654+3A>G	p.? / p.?
6	1974	f	France	CB	**c.297C>A / c.1211T>G**	**p.(Phe99Leu) / p.(Leu404Arg)**
7	1992	f	France	CB	**c.734_745del** / c.1562A>G	**p.(Gly245_Ser248del)** / p.(Tyr521Cys)
8[b]	1988	f	North Africa	CB	c.1277T>C / c.1277T>C	p.(Leu426Pro) / p.(Leu426Pro)
9	1982	m	France	CB	c.209A>C / **c.845delinsAA**	p.(His70Pro) / **p.(Arg282Glnfs*92)**
10[c]	1955	m	Algeria	n/a	**c.526G>A / c.526G>A**	**p.(Glu176Lys) / p.(Glu176Lys)**
11	1986	m	France	CB, later CIE	c.789_791del / **c.1963G>A**	p.(Phe264del) / **p.(Glu655Lys)**
12	1995	f	France	CB	c.1642C>T / **c.1669_1681del**	p.(Arg548Trp) / **p.(Arg558Serfs*2)**
13	1976	f	France	CB	**c.526G>A / c.526G>A**	**p.(Glu176Lys) / p.(Glu176Lys)**
14	1986	f	Italy	CB	c.130_131del / **c.1427A>G**	p.(Asp44Leufs*19) / **p.(Tyr476Cys)**
15[d,e]	1993	f	Italy	CB	c.1261C>T / c.2035C>A	p.(His421Tyr) / p.(Arg679Ser)
16[d,e]	n/a	f	Italy	LI	c.1261C>T / c.1613A>C	p.(His421Tyr) / p.(Gln538Pro)
17[e]	n/a	n/a	Turkey	n/a	c.1180G>A / c.1180G>A	p.(Glu394Lys) / p.(Glu394Lys)
18[b]	n/a	m	Turkey	n/a	c.1734C>A / c.1734C>A	p.(His578Gln) / p.(His578Gln)
19[b]	n/a	f	Turkey	n/a	c.1389del / c.1389del	p.Phe463Leufs*4 / p.Phe463Leufs*4
20	n/a	f	Turkey	n/a	**c.1025T>C / c.1025T>C**	**p.(Leu342Pro) / p.(Leu342Pro)**
21[e]	1993	f	Algeria	n/a	c.632_633del / c.632_633del	p.(Phe211Cysfs*29) / p.(Phe211Cysfs*29)

22	n/a	n/a	Algeria	n/a	c.1579G>A / c.1579G>A	p.(Val527Met) / p.(Val527Met)
23[e]	1982	f	Morocco	CB	c.1078C>G / c.1078C>G	p.(Gln360Glu) / p.(Gln360Glu)
24[e]	n/a	n/a	Algeria	n/a	c.1579G>A / c.1579G>A	p.(Val527Met) / p.(Val527Met)
25	n/a	f	Tunisia	CIE	**c.2064C>G / c.2064C>G**	**p.(Tyr688*) / p.(Tyr688*)**
26	1995	f	Tunisia	CB, later CIE	c.1294C>T / c.1294C>T	p.(Arg432*) / p.(Arg432*)
27	1998	f	France	CIE	**c.845delinsAA / c.1694G>C**	**p.(Arg282Glnfs*92) / p.(Arg565Pro)**
28	1993	f	Denmark	LI	c.1562A>G / c.1562A>G	p.(Tyr521Cys) / p.(Tyr521Cys)
29	n/a	n/a	France	n/a	**c.769C>G / c.769C>G**	**p.(His257Asp) / p.(His257Asp)**
30	n/a	n/a	France	n/a	c.1325G>A / c.1325G>A	p.(Arg442Gln) / p.(Arg442Gln)
31	1994	f	Germany	n/a	**c.325G>T / c.1018del**	**p.(Glu109*) / p.(Leu340Serfs*12)**
32	2005	m	Germany	n/a	**c.47C>T** / c.2012del	**p.(Ser16Leu)** / p.(Gln671Argfs*24)
33	1992	f	Germany	n/a	**c.771_772del / c.771_772del**	**p.(His257Glnfs*116) / p.(His257Glnfs*116)**
34	2002	n/a	Germany	n/a	**c.1609G>A / c.1609G>A**	**p.(Val537Met) / p.(Val537Met)**
35	1972	f	Germany	n/a	c.1562A>G / **c.1861G>T**	p.(Tyr521Cys) / **p.(Asp621Tyr)**
36	2001	n/a	Germany	n/a	c.1148C>T / c.1148C>T	p.(Thr383Met) / p.(Thr383Met)
37	n/a	n/a	Italy	n/a	c.1157G>A / c.1562A>G	p.(Arg386His) / p.(Tyr521Cys)
38	n/a	n/a	Italy	n/a	c.1261C>T / c.1261C>T	p.(His421Tyr) / p.(His421Tyr)
39	n/a	n/a	Italy	n/a	c.1821G>C / **c.371A>T**	p.(Lys607Asn) / **p.(Asp124Val)**
40	n/a	n/a	Italy	n/a	**c.47C>T / c.67T>C**	**p.(Ser16Leu) / p.(Ser23Pro)**
41	n/a	n/a	Italy	n/a	c.1642C>T / c.1642C>T	p.(Arg548Trp) / p.(Arg548Trp)
42	n/a	n/a	Italy	n/a	c.1294C>T / c.1294C>T	p.(Arg432*) / p.(Arg432*)
43[f]	n/a	n/a	Sweden	CIE	c.1562A>G / c.1562A>G	p.(Tyr521Cys) / p.(Tyr521Cys)
44[g]	n/a	n/a	Sweden	CIE	c.1385G>A / c.1385G>A	p.(Gly462Asp) / p.(Gly462Asp)
45[g]	n/a	f	Sweden	SICI	c.199A>T / c.1562A>G	p.(Ile67Phe) / p.(Tyr521Cys)
46[f]	n/a	n/a	Sweden	LI	c.1562A>G / c.1562A>G	p.(Tyr521Cys) / p.(Tyr521Cys)
47[g]	n/a	n/a	Sweden	SICI	c.1562A>G / c.1562A>G	p.(Tyr521Cys) / p.(Tyr521Cys)

48[f]	n/a	n/a	Sweden	LI, CIE	c.1562A>G / c.1562A>G	p.(Tyr521Cys) / p.(Tyr521Cys)
49[g]	n/a	n/a	Sweden	SICI	c.1562A>G / c.1562A>G	p.(Tyr521Cys) / p.(Tyr521Cys)
50[g]	n/a	f	Sweden	LI	c.1579G>A / c.1790C>A	p.(Val527Met) / p.(Ala597Glu)
51[f]	n/a	n/a	Sweden	LI	c.1265C>T / c.1562A>G	p.(Pro422Leu) / p.(Tyr521Cys)
52[g]	n/a	n/a	Denmark	LI	c.1654+3A>G / c.1654+3A>G	p.? / p.?
53[f]	n/a	n/a	Denmark	n/a	c.1654+3A>G / c.1654+3A>G	p.? / p.?
54	2003	f	Austria	n/a	**c.1926+2T>G / c.1926+2T>G**	**p.? / p.?**
55	2010	f	Italy	SICI	c.340C>T / **c.1219G>A**	p.(Arg114Trp) / **p.(Glu407Lys)**
56	2011	m	France	LI	**c.299C>T / c.1655-7C>A**	**p.(Pro100Leu) / p.?**
57	2012	f	Germany	n/a	**c.1495C>T** / c.2036G>T	**p.(Arg499Cys)** / p.(Arg679Leu)
58	2012	m	Germany	n/a	c.1562A>G / c.1192C>T	p.(Tyr521Cys) / p.(His398Tyr)
59	2007	m	Turkey	LI	**c.1594G>A / c.1594G>A**	**p.(Glu532Lys) / p.(Glu532Lys)**
60	2013	w	Ireland	Limited collodion type skin on face	**c.1829C>T / c.1936G>A**	**p.(Thr610Ile) / p.(Gly646Arg)**
61	2009	m	Italy	n/a	c.1163C>T / c.1562A>G	p.(Ala388Val) / p.(Tyr521Cys)
62	1962	f	Italy	n/a	**c.1324C>T / c.1324C>T**	**p.(Arg442Trp) / p.(Arg442Trp)**
63	2007	w	Italy	n/a	**c.938_941dup / c.938_941dup**	**p.(Ala316Profs*59) / p.(Ala316Profs*59)**
64	2012	w	Germany	CB, Erythroderma with pityriasiform desquamation, LI	c.1562A>G / c.1562A>G	p.(Tyr521Cys) / p.(Tyr521Cys)
65	2008	w	Ireland	LI, generalized	**c.1498G>C / c.1977del**	**p.(Asp500His) / p.(Arg660Glyfs*3)**
66	2008	m	Ireland	CB. Later mild fine scaled generalized ichthyosis	c.1405C>T / c.1405C>T	p.(Arg469Trp) / p.(Arg469Trp)
67	1984	w	Turkey	n/a	**c.2041A>T / c.2041A>T**	**p.(Lys681*) / p.(Lys681*)**
68	2014	m	Germany	SICI	**c.1127G>A** / c.1294C>T	**p.(Trp376*)** / p.(Arg432*)
69	2014	m	Ireland	CB with underlying erythroderma	c.1790C>A / **c.2060A>G**	p.(Ala597Glu) / **p.(Tyr687Cys)**

70	2014	m	Germany	CB	**c.1797G>T / c.1797G>T**	**p.(Met599Ile) / p.(Met599Ile)**
71	2014	m	Germany	n/a	c.1562A>G / c.1562A>G	p.(Tyr521Cys) / p.(Tyr521Cys)
72	1997	f	France	SICI	c.340C>T / c.1148C>T	p.(Arg114Trp) / p.(Tyr383Met)
73	2014	f	Germany	CB, later mild erythroderma, pityriasiform desquamation, palmoplantar hyperlinearity	c.1562A>G / c.1562A>G	p.(Tyr521Cys) / p.(Tyr521Cys)
74	2012	f	Germany	CIE	**c.938_941dup** / c.1192C>T	**p.(Ala316Profs*59)** / p.(His398Tyr)
75	2015	f	Germany	CB	c.1156C>T / c.1579G>A	p.(Arg386Cys) / p.(Val257Met)
76	2015	f	Germany	CB with ectropion	c.1562A>G / c.1790C>A	p.(Tyr521Cys) / p.(Ala597Glu)
77	2015	f	Germany	SICI	**c.47C>G / c.208_211dup**	**p.(Ser16Trp) / p.(Lys71Thrfs*55)**
78	2013	m	Germany	SICI, folded earlobe	**c.208_211dup** / c.1790C>A	**p.(Lys71Thrfs*55)** / p.(Ala597Glu)
79	2015	m	Germany	SICI	c.1562A>G / **c.1871C>T**	p.(Tyr521Cys) / **p.(Thr624Ile)**
80	2008	m	Poland/Russia	SICI, deformation of ear	**c.2094C>A / c.2094C>A**	**p.(Ser698Arg) / p.(Ser698Arg)**
81	2015	m	Ireland	Partial collodion membrane, severe intrauterine growth retardation, cardiomyopathy (suspected congenital order of glycosylation)	c.1405C>T / c.1562A>G	p.(Arg469Trp) / p.(Tyr521Cys)
82	1997	f	Germany	n/a	**c.353-2A>G / c.1336_1338del**	**p.? / p.(Leu446del)**
83	2016	m	Italy	CB	c.1192C>T / **c.1937dup**	p.(His398Tyr) / **p.(His647Thrfs*50)**
84	2016	m	Greece	n/a	**c.1015C>G / c.1859C>A**	**p.(Pro339Ala) / p.(Pro620Gln)**
85	2016	m	Germany	SICI	c.1265C>T / c.1265C>T	p.(Pro422Leu) / p.(Pro422Leu)
86	1978	m	Germany	n/a	c.71T>C / c.1654+3A>G	p.(Leu24Pro) / p.?
87	1967	f	Germany	n/a	c.71T>C / **c.1015C>G**	p.(Leu24Pro) / **p.(Pro339Ala)**
88	2012	f	Germany	LI	c.1156C>T / c.1156C>T	p.(Arg386Cys) / p.(Arg386Cys)
89	2017	f	Germany	CB	**c.307C>T** / c.1790C>A	**p.(Gln103*)** / p.(Ala597Glu)
90	1962	f	n/a	n/a	**c.1533-1G>T / c.2005_2037dup**	**p.? / p.(Leu669_Arg679dup)**

91	2000	f	n/a	n/a	c.1630T>C / c.1630T>C	p.(Cys544Arg) / p.(Cys544Arg)
92	1998	m	n/a	n/a	c.1148C>T / c.1790C>A	p.(Thr383Met) / p.(Ala597Glu)
93	1991	m	Poland	no CB	c.1562A>G / c.1562A>G	p.(Tyr521Cys) / p.(Tyr521Cys)
94	2017	m	n/a	CB	c.1163C>T / c.1163C>T	p.(Ala388Val) / p.(Ala388Val)
95	2015	m	n/a	CB	**c.1495C>T / c.1495C>T**	**p.(Arg499Cys) / p.(Arg499Cys)**
96	2006	f	n/a	n/a	**c.928-1G>C** / c.1562A>G	**p.?** / p.(Tyr521Cys)
97	1993	m	Germany	CB, later generalized fine lamellar ichthyosis, generalized Erythroderma	**c.1336_1338del** / c.1562A>G	**p.(Leu446del)** / p.(Tyr521Cys)
98	1995	m	Germany	Mild Erythroderma with mild lamellar ichthyosis, mild PPK	**c.698G>A** / c.1562A>G	**p.(Trp233*)** / p.(Tyr521Cys)
99	2013	m	Syria	CIE at birth with folded earlobe and strong lichenification, mild erythroderma	c.1579G>A / c.1579G>A	p.(Val527Met) / p.(Val527Met)
100	1989	m	Germany	CB, ectropion and eclabion, improving (SICI)	c.1790C>A / c.1790C>A	p.(Ala597Glu) / p.(Ala597Glu)
101	2004	m	Serbia	CB, later mild erythroderma with palmar hyperkeratosis	c.1562A>G / c.1562A>G	p.(Tyr521Cys) / p.(Tyr521Cys)
102	2019	m	Germany	Ectropion ab birth	c.1562A>G / c.1562A>G	p.(Tyr521Cys) / p.(Tyr521Cys)
103	2019	m	Germany	n/a	c.1562A>G / c.1562A>G	p.(Tyr521Cys) / p.(Tyr521Cys)
104	2019	f	Germany	CB, improving (SICI)	**c.1258T>G** / c.1562A>G	**p.(Cys420Gly)** / p.(Tyr521Cys)
105	2019	m	n/a	CB	c.1562A>G / c.1790C>A	p.(Tyr521Cys) / p.(Ala597Glu)
106	1990	f	Germany	no CB, fine lamellar ichthyosis from birth	c.340C>T / c.1562A>G	p.(Arg114Trp) / p.(Tyr521Cys)
107	2007	m	n/a	n/a	**c.341G>A / c.341G>A**	**p.(Arg114Gln) / p.(Arg114Gln)**

108[h]	2006	m	Turkey	CB, later CIE, improved with age	c.1463G>A / c.1463G>A	p.(Arg488His) / p.(Arg488His)
109[h]	2010	f	Turkey	CB, later CIE	c.1463G>A / c.1463G>A	p.(Arg488His) / p.(Arg488His)
110[h]	2016	f	Serbia	CB, later CIE	c.1562A>G / c.1562A>G	p.(Tyr521Cys) / p.(Tyr521Cys)
111[h]	2017	m	Austria	CB, later CIE	c.1562A>G / c.1562A>G	p.(Tyr521Cys) / p.(Tyr521Cys)
112[h]	1988	m	Austria	CB, later LI	c.467_470dup / **c.938T>C**	p.(His158Cysfs*20) / **p.(Ile313Thr)**
113[h]	1995	f	Austria	CB, later CIE	c.1562A>G / **c.1787C>T**	p.(Tyr521Cys) / **p.(Pro596Leu)**
114[h]	2018	f	Austria	CB, later CIE	**c.1676C>T** / c.1562A>G	**p.(Thr559Ile)** / p.(Tyr521Cys)
115[h]	2016	f	Turkey	CB, later CIE	c.1071+1dup / c.1071+1dup	p.? / p.?
116[h]	2019	f	Austria	CB, later CIE	c.1562A>G / c.1790C>A	p.(Tyr521Cys) / p.(Ala597Glu)
117[i]	1981	m	Kurdish	n/a	**c.195_220del26 / c.195_220del26**	**p.(lle66Argfs*50) / p.(lle66Argfs*50)**
118[j]	1986	m	Finland	CB	c.1562A>G / c.1562A>G	p.(Tyr521Cys) / p.(Tyr521Cys)
119[j]	1989	f	Ukraine	No CB, only erythroderma	c.1562A>G / c.1562A>G	p.(Tyr521Cys) / p.(Tyr521Cys)
120[j]	2009	m	Russia	n/a	c.1562A>G / c.1790C>A	p.(Tyr521Cys) / p.(Ala597Glu)
121[k]	2006	f	France	CB	c.1350del / c.1562A>G	p.(Leu451Serfs*16) / p.(Tyr521Cys)
122[k]	2005	m	France	CB	c.1562A>G / **c.2036G>A**	p.(Tyr521Cys) / **p.(Arg679His)**
123[k]	2011	F	France	CB	**c.1634T>G / c.1742T>G**	**p.(Leu545Arg) / p.(Val581Gly)**
124[k]	1995	F	Romania	CB	c.1562A>G / c.1790C>A	p.(Tyr521Cys) / p.(Ala597Glu)
125[k]	1995	n/a	Romania	CB	**c.1071+1G>C** / c.1562A>G	**p.?** / p.(Tyr521Cys)
126[k]	1983	f	Algeria/France	CB	**c.58G>T** / c.1463G>A	**p.(Asp20Tyr)** / p.(Arg488His)
127[k]	2017	f	France	CB	**c.371A>T / c.864del**	**p.(Asp124Val) / p.(Val289Serfs*63)**
128[k]	2015	m	Turkey	CB	**c.793G>A** / c.1562A>G	**p.(Gly265Arg)** / p.(Tyr521Cys)
129[k]	2018	f	France	CB	**c.893T>C / c.893T>C**	**p.(Leu298Pro) / p.(Leu298Pro)**
130[l]	2014	f	Spain	n/a	c.1272dup / c.1272dup	p.(Lys425Glnfs*24) / p.(Lys425Glnfs*24)
131[l]	2012	f	Spain	n/a	c.1272dup / **c.1594G>A**	p.(Lys425Glnfs*24) / **p.(Glu532Lys)**
132[l]	2015	f	Spain	n/a	**c.416_417del / c.416_417del**	**p.(Ala139Glufs*37) / p.(Ala139Glufs*37)**
133[l]	2007	f	Spain	n/a	c.1562A>G / **c.814G>T**	p.(Tyr521Cys) / **p.(Val272Phe)**

134[l]	1975	m	Spain	n/a	**c.1732C>T / c.1732C>T**	**p.(His578Tyr) / p.(His578Tyr)**
135[m]	1995	f	Macedonia	CB	**c.1349G>A / c.1369T>C**	**p.(Gly450Glu) / p.(Ser457Pro)**
136[m]	2015	m	Italy	CB	**c.353-2A>G** / c.527+1G>A	**p.?** / p.?
137[m]	2005	n/a	Italy	CB	**c.47C>T / c.47C>T**	**p.(Ser16Leu) / p.(Ser16Leu)**
138[m,n]	2008	n/a	Italy	CB	**c.47C>T / c.47C>T**	**p.(Ser16Leu) / p.(Ser16Leu)**
139[m]	2007	n/a	Italy	CB	**c.47C>T / c.938_941dup**	**p.(Ser16Leu) / p.(Ala316Profs*59)**
140[m]	2010	n/a	Italy	CB	c.1562A>G / **c.1654+1G>A**	p.(Tyr521Cys) / **p.?**
141[m]	2013	n/a	Italy	CB	**c.353-2A>G / c.1057C>A**	**p.? / p.(Pro353Thr)**

[a,d]two branches of the same family, [b]previously published in Jobard *et al*. (2002), [c]patient is identical with patient 5 in Table S2, [e]previously published in Leseur *et al*. (2007), [f]previously published in Pigg *et al*. (2016), [g]previously published in Vahlquist *et al*. (2010), [h]patients contributed by R. G. and M. S., [i]patient contributed by v. d. A. and M. C. B., [j]patients contributed by K. H.-M., [k]patients contributed by J. M.-H. and N. J., [l]patients contributed by A. H.-M., [m]patients contributed by S. G. and M. B., [n]this patient carries additionally a 22qter deletion. In bold: novel mutations, CB: collodion baby, LI: lamellar ichthyosis, SICI: self-improving collodion ichthyosis, PPK: palmoplantar hyperkeratosis

Table S2: Identified mutations in *ALOXE3* in patients with ARCI

Patient	Year of birth	Sex	Origin	Phenotype	Nucleotid change	Consequences of mutation (amino acid level)
1	1984	f	France	n/a	c.1889C>T / c.1889C>T	p.(Pro630Leu) / p.(Pro630Leu)
2[a]	n/a	n/a	Turkey	n/a	c.1498G>T / c.1498G>T	p.(Val500Phe) / p.(Val500Phe)
3[a]	1977	m	France	n/a	c.700C>T / c.700C>T	p.(Arg234*) / p.(Arg234*)
4	1986	m	France	n/a	c.1889C>T / c.1889C>T	p.(Pro630Leu) / p.(Pro630Leu)
5[b]	1955	m	Algeria	n/a	**c.1193C>T / c.1193C>T**	**p.(Ser398Phe) / p.(Ser398Phe)**
6	1958	f	France	n/a	**c.1786-2A>G** / c.1889C>T	**p.?** / p.(Pro630Leu)
7[a]	1993	f	Morocco	n/a	c.1186C>A / c.1186C>A	p.(Arg396Ser) / p.(Arg396Ser)
8	n/a	m	Italy	n/a	**c.758del** / c.1889C>T	**p.(Phe253Serfs*27)** / p.(Pro630Leu)
9	1984	f	Tunisia	n/a	**c.957G>A / c.957G>A**	**p.(Glu319=) / p.(Glu319=)**
10	2000	f	Denmark	n/a	c.327C>A / c.1889C>T	p.(Cys109*) / p.(Pro630Leu)
11	1935	f	Germany	LI	**c.1803_1804dup** / c.700C>T	**p.(Met602Asnfs*30)** / p.(Arg234*)
12	1995	f	Germany	n/a	**c.1786-63_1807del / c.1786-63_1807del**	**p.? / p.?**
13	1967	f	Germany	LI	c.700C>T / c.700C>T	p.(Arg234*) / p.(Arg234*)
14	1988	m	Germany	n/a	c.700C>T / c.700C>T	p.(Arg234*) / p.(Arg234*)
15[c]	n/a	n/a	Sweden	n/a	c.353-1G>C / c.1889C>T	p.? / p.(Pro630Leu)
16[c]	n/a	n/a	Sweden	n/a	c.631C>T / c.1889C>T	p.(Arg211*) / p.(Pro630Leu)
17[c,d]	n/a	n/a	Sweden	n/a	c.1280T>C / c.1280T>C	p.(Leu427Pro) / p.(Leu427Pro)
18[c,d]	n/a	m	Sweden	n/a	c.700C>T / c.1889C>T	p.Arg234*/ p.(Pro630Leu)
19[c]	n/a	n/a	Sweden	n/a	c.1305+1_1305+2delinsTA / c.1305+1_1305+2delinsTA	p.? / p.?
20[c]	1967	f	Denmark	LI/CIE	c.1889C>T / c.1889C>T	p.(Pro630Leu) / p.(Pro630Leu)
21[c]	1999	f	Denmark	CIE	c.700C>T / c.1889C>T	p.(Arg234*) / p.(Pro630Leu)
22[c]	1973	f	Denmark	CIE	c.631C>T / c.1889C>T	p.(Arg211*) / p.(Pro630Leu)

23[c]	1985	f	Denmark	LI/CIE	c.327C>A / c.1889C>T	p.(Cys109*) / p.(Pro630Leu)
24	2010	m	Germany	SICI	c.700C>T / c.1889C>T	p.(Arg234*) / p.(Pro630Leu)
24	1997	f	Germany	SICI	c.1889C>T / c.1889C>T	p.(Pro630Leu) / p.(Pro630Leu)
26	2010	f	n/a	no CB, erythroderma and desquamation at birth	c.38_41del / **c.952dup**	p.(Tyr13*) / **p.(Leu318Profs*58)**
27	2006	m	Germany	SICI	**c.57_63del** / c.1889C>T	**p.(Asp20Serfs*17)** / p.(Pro630Leu)
28	2013	m	Germany	SICI	**c.397A>G / c.1812T>A**	**p.(Arg133Gly) / p.(Asn604Lys)**
29	2003	m	France	n/a	**c.271G>T / c.271G>T**	**p.(Glu91*) / p.(Glu91*)**
30	2009	f	Germany	n/a	c.1889C>T / c.1889C>T	p.(Pro630Leu) / p.(Pro630Leu)
31	2012	f	Germany	n/a	c.1889C>T / c.1889C>T	p.(Pro630Leu) / p.(Pro630Leu)
32	1992	f	France	no CB	c.1889C>T / c.1889C>T	p.(Pro630Leu) / p.(Pro630Leu)
33	2008	m	Germany	Fine white ichthyosis, bend in the ear, short fingers	c.700C>T c.700C>T	p.(Arg234*) / p.(Arg234*)
34	2007	f	Germany	SICI	c.1889C>T / c.1889C>T	p.(Pro630Leu) / p.(Pro630Leu)
35	1984	f	France	CB	**c.957G>A / c.957G>A**	**p.(Glu319=) / p.(Glu319=)**
36	2014	f	n/a	blisters at birth	**c.680+1G>A / c.680+1G>A**	**p.? / p.?**
37	2017	m	Germany	CB	**c.1812T>A** / c.1889C>T	**p.(Asn604Lys)** / p.(Pro630Leu)
38	2017	f	Germany	n/a	**c.1061G>A** / c.1889C>T	**p.(Trp354*)** / p.(Pro630Leu)
39	1963	m	Germany	n/a	c.1889C>T / c.1889C>T	p.(Pro630Leu) / p.(Pro630Leu)
40	1987	f	Germany	n/a	**c.833A>G / c.1292dup**	**p.(Tyr278Ser) / p.(His431Glnfs*90)**
41	1988	f	Germany	n/a	c.1889C>T / c.1889C>T	p.(Pro630Leu) / p.(Pro630Leu)
42	2007	m	Germany	n/a	c.1889C>T / c.1889C>T	p.(Pro630Leu) / p.(Pro630Leu)
43	1977	f	Germany	n/a	c.700C>T / c.1889C>T	p.(Arg234*) / p.(Pro630Leu)
44	2004	f	Germany	n/a	c.700C>T / c.1889C>T	p.(Arg234*) / p.(Pro630Leu)
45	1992	f	Germany	n/a	c.1889C>T / c.1889C>T	p.(Pro630Leu) / p.(Pro630Leu)
46	1980	m	Germany	n/a	c.631C>T / c.1031_1039del	p.(Arg211*) / p.(Gln344_Ala347delinsPro)

47	1994	m	Germany	n/a	c.38_41del / c.700C>T	p.(Tyr13*) / p.(Arg234*)
48	1994	f	Germany	CIE at birth, no CB	c.1889C>T / c.1889C>T	p.(Pro630Leu) / p.(Pro630Leu)
49	2017	m	Germany	no CB, initial diagnosis IV	c.631C>T / c.1889C>T	p.(Arg211*) / p.(Pro630Leu)
50	2012	m	Germany	no CB, initial diagnosis IV/XLI	c.1889C>T / c.1889C>T	p.(Pro630Leu) / p.(Pro630Leu)
51	2018	m	Germany	n/a	c.700C>T / c.700C>T	p.(Arg234*) / p.(Arg234*)
52	2012	m	Germany	no CB, initial diagnosis XLI	c.700C>T / c.1889C>T	p.(Arg234*) / p.(Pro630Leu)
53	1998	f	Germany	no CB, initial diagnosis IV	**c.1061G>A** / c.1889C>T	**p.(Trp354*)** / p.(Pro630Leu)
54	1995	f	Germany	n/a	**c.1292dup** / c.1889C>T	**p.(His431Glnfs*90)** / p.(Pro630Leu)
55	1996	f	Germany	no CB, erythroderma and desquamation at birth	c.1889C>T / c.1889C>T	p.(Pro630Leu) / p.(Pro630Leu)
56	1992	f	Romania	SICI	**c.923T>C** / c.1889C>T	**p.(Leu308Pro)** / p.(Pro630Leu)
57	2010	f	Germany	CB, later mild erythroderma and mild desquamation	c.700C>T / c.1889C>T	p.(Arg234*) / p.(Pro630Leu)
58	2016	m	Ukraine	LI at birth	c.700C>T / c.700C>T	p.(Arg234*) / p.(Arg234*)
59	1989	w	Germany	n/a	c.700C>T / c.700C>T	p.(Arg234*) / p.(Arg234*)
60	1987	m	Northern Europe	CB	c.1889C>T / c.1889C>T	p.(Pro630Leu) / p.(Pro630Leu)
61	2018	f	Germany	n/a	c.700C>T / c.1889C>T	p.(Arg234*) / p.(Pro630Leu)
62	2020	m	Germany	SICI	c.700C>T / c.1889C>T	p.(Arg234*) / p.(Pro630Leu)
63	1972	f	Germany	n/a	c.1889C>T / c.1889C>T	p.(Pro630Leu) / p.(Pro630Leu)
64[e]	1981	m	Turkey	CIE, no CB	**c.1954C>T / c.1954C>T**	**p.(Gln652*) / p.(Gln652*)**
65[e]	2012	m	Austria	CIE, no CB	c.700C>T / c.1889C>T	p.(Arg234*) / p.(Pro630Leu)
66[e]	2017	f	Austria	CIE, no CB	**c.1031A>C / c.1031A>C**	**p.(Gln344Pro) / p.(Gln344Pro)**

67[e]	1971	m	Austria	CB	c.700C>T / c.700C>T	p.(Arg234*) / p.(Arg234*)
68[e]	1959	f	Austria	CIE, no CB	**c.1937_1944del / c.1937_1944del**	**p.(Ser646Thrfs*13) / p.(Ser646Thrfs*13)**
69[e]	1988	f	Austria	Mild CB, generalized fine to medium scaling	c.700C>T / c.1889C>T	p.(Arg234*) / p.(Pro630Leu)
70[e]	2014	f	Austria	CIE, no CB	c.700C>T / c.1889C>T	p.(Arg234*) / p.(Pro630Leu)
71[e]	2000	f	Turkey	CIE, no CB	**c.1393-1G>A / c.1393-1G>A**	**p.? / p.?**
72[e]	2002	m	Turkey	CIE, no CB	**c.1393-1G>A / c.1393-1G>A**	**p.? / p.?**
73[e]	1995	f	Austria	CIE, no CB	**c.308A>C** / c.700C>T	**p.(Gln103Pro)** / p.(Arg234*)
74[f]	2014	f	Netherlands	CB	**c.1246T>C** / c.1889C>T	**p.(Cys416Arg)** / p.(Pro630Leu)
75[f]	1990	f	Netherlands	CB	c.1889C>T / c.1889C>T	p.(Pro630Leu) / p.(Pro630Leu)
76[f]	1984	f	Netherlands	CB	c.700C>T / c.1889C>T	p.(Arg234*) / p.(Pro630Leu)
77[g]	2013	m	Finland	Erythroderma, no CB	c.700C>T / c.1889C>T	p.(Arg234*) / p.(Pro630Leu)
78[h]	1961	f	France	Erythroderma, no CB	c.700C>T / c.700C>T	p.(Arg234*) / p.(Arg234*)
79[h]	1992	f	France	Erythroderma, no CB	**c.1164G>T / c.1164G>T**	**p.(Trp388Cys) / p.(Trp388Cys)**
80[h]	1991	f	France	Erythroderma, no CB	**c.1202T>A** / c.1889C>T	**p.(Leu401Gln)** / p.(Pro630Leu)
81[h]	2012	m	Morroco	CB	c.1186C>A / c.1186C>A	p.(Arg396Ser) / p.(Arg396Ser)
82[h]	2013	m	Turkey	Erythroderma, no CB	c.1208A>G / c.1208A>G	p.(His403Arg) / p.(His403Arg)
83[i]	2017	f	Spain	n/a	c.1889C>T / **deletion exon 15**	p.(Pro630Leu) / **p.?**

[a]previously published in Jobard *et al.* (2002), [b]patient is identical with patient 10 in Table S1, [c]previously published in Hellström Pigg *et al.* (2016), [d]previously published in Vahlquist *et al.* (2010), [e]patients contributed by R. G. and M. S., [f]patients contributed by v. d. A. and M. C. B., [g]patient contributed by K. H.-J., [h]patients contributed by J. M.-H. and N. J., [i]patient contributed by A. H.-M.. In bold: novel mutations, LI: lamellar ichthyosis, CIE: congenital ichthyosiform erythroderma, SICI: self-improving collodion baby, CB: collodion baby, IV: ichthyosis vulgaris, XLI: X-linked ichthyosis

Table S3: Results of bioinformatic tools for novel mutations in *ALOX12B*

**Mutation**		**gnomAD**	**rs number**	**ClinVar**	**MutationTaster**	**Polyphen-2 (HumVar)**	**Provean (cutoff=-2.5)**	**SIFT (cutoff=0.05)**	**fathmm**	**NNSplice**	**NetGene2**	**SSP (VarSEAK)**
**c.47C>G**	**p.(Ser16Trp)**	n/a	n/a	n/a	polymorphism (0.995)	probably damaging (0.988)	deleterious (-4.20)	damaging (0.000)	tolerated (-1.13)	-	-	-
**c.47C>T**	**p.(Ser16Leu)**	0x homo, 3x het, MAF 0.001%	rs147784568	n/a	polymorphism (1.0)	possibly damaging (0.531)	deleterious (-2.64)	damaging (0.001)	tolerated (-1.11)	-	-	-
**c.58G>T**	**p.(Asp20Tyr)**	n/a	n/a	n/a	polymorphism (1.0)	probably damaging (0.919)	deleterious (-6.60)	damaging (0.001)	tolerated (-1.24)	-	-	-
**c.67T>C**	**p.(Ser23Pro)**	n/a	n/a	n/a	disease causing (0.552)	probably damaging (0.992)	deleterious (-3.47)	damaging (0.032)	tolerated (-1.12)	-	-	-
**c.195_220del**	**p.(Ile66Argfs*50)**	n/a	n/a	n/a	disease causing (1.0)	-	-	-	-	-	-	-
**c.208_211dup**	**p.(Lys71Thrfs*55)**	n/a	n/a	n/a	disease causing (1.0)	-	-	-	-	-	-	-
**c.297C>A**	**p.(Phe99Leu)**	n/a	n/a	n/a	disease causing (1.0)	probably damaging (0.960)	deleterious (-5.59)	damaging (0.002)	tolerated (-1.23)	-	-	-
**c.299C>T**	**p.(Pro100Leu)**	n/a	rs1256507861	n/a	disease causing (1.0)	probably damaging (0.992)	deleterious (-9.31)	damaging (0.000)	tolerated (-0.25)	-	-	-
**c.307C>T**	**p.(Gln103*)**	n/a	n/a	n/a	disease causing (1.0)	-	-	-	-	-	-	-
**c.325G>T**	**p.(Glu109*)**	n/a	n/a	n/a	disease causing (1.0)	-	-	-	-	-	-	-
**c.341G>A**	**p.(Arg114Gln)**	0x homo, 2x het, MAF 0.0008%	rs745541957	n/a	disease causing (0.661)	possibly damaging (0.532)	neutral (-2.42)	tolerated (2.81)	damaging (-2.60)	-	-	-
**c.353-2A>G**	**p.?**	0x homo, 1x het, MAF 0.0004%	rs775524204	1x likely pathogenic	-	-	-	-	-	Loss of acceptor splice site	Loss of acceptor splice site	No AG, loss of function for authentic splice site
**c.371A>T**	**p.(Asp124Val)**	0x homo, 2x het, MAF 0.0008%	rs138503921	n/a	disease causing (0.676)	possibly damaging (0.697)	deleterious (-6.58)	damaging (0.000)	damaging (-2.79)	-	-	-
**c.416_417del**	**p.(Ala139Glufs*37)**	n/a	n/a	n/a	disease causing (1.0)	-	-	-	-	-	-	-
**c.526G>A**	**p.(Glu176Lys)**	0x homo, 95x het, MAF 0.03%	rs149039053	1x uncertain significance	disease causing (0.635)	benign (0.034)	neutral (-0.13)	tolerated (0.108)	damaging (-2.54)	No splicing effect (donor site 0.66 wt → 0.90 mut)	No splicing effect	No splicing effect (Δ+34.78%)
**c.698G>A**	**p.(Trp233*)**	n/a	n/a	n/a	disease causing (1.0)	-	-	-	-	-	-	-

**c.734_745del**	**p.(Gly245_Ser248del)**	n/a	n/a	n/a	-	-	-	-	-	-	-	-
**c.769C>G**	**p.(His257Asp)**	0x homo, 1x het, MAF 0.0004%	rs1233776328	n/a	disease causing (0.996)	probably damaging (0.993)	deleterious (-8.27)	damaging (0.004)	damaging (-2.54)	-	-	-
**c.771_772del**	**p.(His257Glnfs*116)**	n/a	n/a	n/a	-	-	-	-	-	-	-	-
**c.793G>A**	**p.(Gly265Arg)**	n/a	n/a	n/a	disease causing (1.0)	probably damaging (0.998)	deleterious (-5.66)	damaging (0.001)	damaging (-2.86)	-	-	-
**c.814G>T**	**p.(Val272Phe)**	n/a	n/a	n/a	polymorphism (1.0)	possibly damaging (0.732)	deleterious (-2.83)	damaging (0.009)	tolerated (-1.23)	-	-	-
**c.845delinsAA**	**p.(Arg282Glnfs*92)**	n/a	n/a	n/a	-	-	-	-	-	-	-	-
**c.864del**	**p.(Val289Serfs*63)**	n/a	n/a	n/a	-	-	-	-	-	-	-	-
**c.893T>C**	**p.(Leu298Pro)**	n/a	n/a	n/a	disease causing (1.0)	probably damaging (1.000)	deleterious (-5.69)	damaging (0.000)	damaging (-1.83)	-	-	-
**c.928-1G>C**	**p.?**	n/a	n/a	n/a	-	-	-	-	-	Loss of acceptor splice site	Loss of acceptor splice site	No AG, loss of function for authentic splice site
**c.938T>C**	**p.(Ile313Thr)**	n/a	rs1197603391	n/a	disease causing (1.0)	probably damaging (0.987)	deleterious (-4.13)	damaging (0.001)	damaging (-2.84)	-	-	-
**c.938_941dup**	**p.(Ala316Profs*59)**	n/a	n/a	n/a	disease causing (1.0)	-	-	-	-	-	-	-
**c.1015C>G**	**p.(Pro339Ala)**	n/a	n/a	1x uncertain significance	disease causing (1.0)	probably damaging (0.999)	deleterious (-7.48)	damaging (0.005)	damaging (-2.86)	-	-	-
**c.1018del**	**p.(Leu340Serfs*12)**	n/a	n/a	n/a	-	-	-	-	-	-	-	-
**c.1025T>C**	**p.(Leu342Pro)**	n/a	n/a	n/a	disease causing (1.0)	probably damaging (1.000)	deleterious (-6.44)	damaging (0.000)	tolerated (-1.47)	-	-	-
**c.1057C>A**	**p.(Pro353Thr)**	n/a	n/a	n/a	disease causing (1.0)	probably damaging (0.999)	deleterious (-7.42)	damaging (0.000)	damaging (-4.55)	-	-	-
**c.1071+1G>C**	**p.?**	n/a	n/a	n/a	-	-	-	-	-	-	Loss of donor splice site	No GT, loss of function for authentic splice site
**c.1127G>A**	**p.(Trp376*)**	n/a	n/a	n/a	disease causing (1.0)	-	-	-	-	-	-	-
**c.1211T>G**	**p.(Leu404Arg)**	n/a	n/a	n/a	disease causing (1.0)	probably damaging (0.998)	deleterious (-5.50)	damaging (0.000)	tolerated (-1.23)	-	-	-

**c.1219G>A**	**p.(Glu407Lys)**	n/a	n/a	n/a	disease causing (1.0)	probably damaging (0.998)	deleterious (-1.99)	damaging (0.000)	damaging (-3.79)	-	-	-
**c.1258T>G**	**p.(Cys420Gly)**	n/a	rs1598178838	1x uncertain significance	disease causing (0.968)	benign (0.345)	deleterious (-8.51)	tolerated (0.159)	tolerated (-1.00)	-	-	-
**c.1309A>T**	**p.(Ile437Phe)**	n/a	n/a	n/a	disease causing (1.0)	probably damaging (0.991)	deleterious (-3.46)	damaging (0.001)	damaging (-3.34)	-	-	-
**c.1324C>T**	**p.(Arg442Trp)**	0x homo, 4x het, MAF 0.002%	rs764862348	n/a	disease causing (1.0)	probably damaging (1.000)	deleterious (-6.92)	damaging (0.000)	damaging (-2.16)	-	-	-
**c.1336_1338del**	**p.(Leu446del)**	n/a	n/a	n/a	-	-	-	-	-	-	-	-
**c.1349G>A**	**p.(Gly450Glu)**	0x homo, 1x het, MAF 0.0005%	rs772257172	n/a	disease causing (1.0)	probably damaging (0.994)	deleterious (-5.09)	damaging (0.008)	damaging (-2.64)	-	-	-
**c.1369T>C**	**p.(Ser457Pro)**	n/a	n/a	n/a	polymorphism (1.0)	benign (0.114)	deleterious (-2.90)	tolerated (0.161)	damaging (-2.66)	-	-	-
**c.1427A>G**	**p.(Tyr476Cys)**	n/a	n/a	n/a	disease causing (1.0)	probably damaging (1.000)	deleterious (-8.54)	damaging (0.000)	damaging (-3.00)	-	-	-
**c.1495C>T**	**p.(Arg499Cys)**	0x homo, 4x het, MAF 0.002%	rs368323729	n/a	disease causing (1.0)	probably damaging (0.999)	deleterious (-7.69)	damaging (0.000)	tolerated (-1.10)	-	-	-
**c.1498G>C**	**p.(Asp500His)**	n/a	rs1064796312	1x uncertain significance	disease causing (0.997)	possibly damaging (0.835)	deleterious (-6.19)	damaging (0.036)	damaging (-2.76)	-	-	-
**c.1533-1G>T**	**p.?**	n/a	n/a	n/a	-	-	-	-	-	-	Loss of acceptor splice site	No AG, loss of function for authentic splice site
**c.1594G>A**	**p.(Glu532Lys)**	0x homo, 1x het, MAF 0.0004%	rs768093512	n/a	disease causing (1.0)	probably damaging (1.000)	deleterious (-3.84)	damaging (0.001)	damaging (-2.00)	-	-	-
**c.1609G>A**	**p.(Val537Met)**	n/a	rs962267153	n/a	disease causing (0.990)	possibly damaging (0.897)	neutral (-1.95)	tolerated (0.147)	tolerated (-1.12)	-	-	-
**c.1634T>G**	**p.(Leu545Arg)**	n/a	n/a	n/a	polymorphism (0.999)	probably damaging (0.934)	deleterious (-3.47)	damaging (0.001)	tolerated (-1.15)	-	-	-
**c.1654+1G>A**	**p.?**	n/a	n/a	n/a	-	-	-	-	-	Loss of donor splice site	Loss of donor splice site	No GT, loss of function for authentic splice site
**c.1655-7C>A**	**p.?**	n/a	n/a	n/a	-	-	-	-	-	Novel acceptor splice site at position c.1655-7 (0.96)	Novel acceptor splice site at position c.1655-7 (0.00)	Position c.1655-7: De novo AG in AG-Exclusion-Zone. Likely loss of function for authentic splice site
**c.1669_1681del**	**p.(Arg558Serfs*2)**	n/a	n/a	n/a	-	-	-	-	-	-	-	-

**c.1676C>T**	**p.(Thr559Ile)**	n/a	n/a	n/a	disease causing (0.999)	probably damaging (0.958)	deleterious (-5.54)	damaging (0.001)	damaging (-2.65)	-	-	-
**c.1694G>C**	**p.(Arg565Pro)**	n/a	n/a	n/a	polymorphism (0.999)	benign (0.025)	deleterious (-3.32)	damaging (0.002)	damaging (-2.58)	-	-	-
**c.1732C>T**	**p.(His578Tyr)**	n/a	n/a	n/a	disease causing (1.0)	probably damaging (0.995)	deleterious (-5.99)	damaging (0.000)	damaging (-6.52)	-	-	-
**c.1742T>G**	**p.(Val581Gly)**	n/a	n/a	n/a	disease causing (1.0)	probably damaging (0.999)	deleterious (-6.97)	damaging (0.000)	damaging (-1.76)	-	-	-
**c.1787C>T**	**p.(Pro596Leu)**	0x homo, 1x het, MAF 0.003%	rs1360781582	n/a	disease causing (1.0)	probably damaging (0.993)	deleterious (-9.96)	damaging (0.001)	damaging (-4.50)	-	-	-
**c.1797G>T**	**p.(Met599Ile)**	n/a	n/a	n/a	disease causing (1.0)	possibly damaging (0.791)	deleterious (-3.32)	damaging (0.007)	damaging (-2.63)	-	-	-
**c.1829C>T**	**p.(Thr610Ile)**	0x homo, 5x het, MAF 0.002%	rs773052129	n/a	polymorphism (0.963)	possibly damaging (0.772)	deleterious (-3.11)	damaging (0.004)	tolerated (-1.04)	-	-	-
**c.1859C>A**	**p.(Pro620Gln)**	0x homo, 1x het, MAF 0.0004%	rs780420901	1x pathogenic	disease causing (1.0)	probably damaging (1.000)	deleterious (-7.63)	damaging (0.001)	damaging (-3.45)	-	-	-
**c.1861G>T**	**p.(Asp621Tyr)**	n/a	n/a	n/a	polymorphism (0.995)	probably damaging (0.950)	deleterious (-4.84)	damaging (0.021)	damaging (-2.69)	-	-	-
**c.1871C>T**	**p.(Thr624Ile)**	n/a	n/a	n/a	disease causing (0.967)	benign (0.158)	neutral (-1.44)	tolerated (0.435)	damaging (-2.58)	-	-	-
**c.1926+2T>G**	**p.?**	n/a	n/a	n/a	-	-	-	-	-	-	-	-
**c.1936G>A**	**p.(Gly646Arg)**	0x homo, 2x het, MAF 0.0008%	rs1398340320	n/a	disease causing (1.0)	probably damaging (1.000)	deleterious (-7.94)	damaging (0.001)	damaging (-2.66)	-	-	-
**c.1937dup**	**p.(His647Thrfs*50)**	n/a	n/a	n/a	-	-	-	-	-	-	-	-
**c.1963G>A**	**p.(Glu655Lys)**	n/a	n/a	n/a	disease causing (1.0)	probably damaging (0.936)	deleterious (-3.86)	damaging (0.002)	damaging (-2.69)	-	-	-
**c.1977del**	**p.(Arg660Glyfs*3)**	0x homo, 1x het, MAF 0.0004%	rs1230917852	n/a	-	-	-	-	-	-	-	-
**c.2000A>C**	**p.(Gln667Pro)**	n/a	n/a	n/a	polymorphism (1.0)	benign (0.034)	neutral (-2.00)	tolerated (0.121)	damaging (-2.61)	-	-	-
**c.2005_2037dup**	**p.(Leu669_Arg679dup)**	n/a	n/a	n/a	-	-	-	-	-	-	-	-
**c.2021_2022dup**	**p.(Asp675Thrfs*21)**	n/a	n/a	n/a	-	-	-	-	-	-	-	-

**c.2036G>A**	**p.(Arg679His)**	0x homo, 1x het, MAF 0.0004%	rs397514528	n/a	disease causing (1.0)	probably damaging (0.999)	deleterious (-4.80)	damaging (0.000)	damaging (-1.50)	-	-	-
**c.2041A>T**	**p.(Lys681*)**	n/a	n/a	n/a	disease causing (1.0)	-	-	-	-	-	-	-
**c.2060A>G**	**p.(Tyr687Cys)**	0x homo, 1x het, MAF 0.0004%	rs1482844053	n/a	disease causing (1.0)	probably damaging (1.000)	deleterious (-8.64)	damaging (0.000)	damaging (-4.08)	-	-	-
**c.2064C>G**	**p.(Tyr688*)**	n/a	n/a	n/a	disease causing (1.0)	-	-	-	-	-	-	-
**c.2094C>A**	**p.(Ser698Arg)**	n/a	n/a	n/a	disease causing (1.0)	probably damaging (0.999)	deleterious (-4.80)	damaging (0.000)	damaging (-3.18)	-	-	-

n/a: not listed in the database. homo: homozygous. Het: heterozygous. MAF: minor allele frequency. -: not evaluated. wt: wildtype, mut: mutation. Scores are in brackets.
gnomAD: The data set provided on this website spans 125,748 exome sequences and 15,708 whole-genome sequences from unrelated individuals sequenced as part of various disease-specific and population genetic studies.
Mutation Taster: The probability value is the probability of the prediction. A value close to 1 indicates a high security of the prediction.
PolyPhen-2: HumVar prediction, the score ranges from 1.0 (damaging) to 0.0 (benign).
Provean: Cutoff threshold is -2.5. Variants with a score equal to or below -2.5 are considered "deleterious", variants with a score above -2.5 are considered "neutral".
SIFT: Cutoff threshold is 0.05. Substitutions with less than 0.05 are predicted as damaging.
Fathmm: Prediction algorithm: weighted. Scores approximately equal to zero indicate no significant change in the underlying amino acid probabilities, scores less than zero indicate an unfavorable substitution, scores greater than zero indicate a favorable substitution.
NNSplice: Splice site predictions for 1 sequence with donor score cutoff 0.40, acceptor score cutoff 0.40. Score ranging from 0 to 1, higher score implies a more potential splice site.
NetGene2: Cutoff values used for confidence: highly confident donor sites (H): 95.0 %, nearly all true donor sites: 50.0 %, highly confident acceptor sites (H): 95.0 %, nearly all true acceptor sites: 20.0 %. Confidence score ranging from 0 to 1, higher score implies a higher confidence of true site.
SSP (VarSEAK): Score: Prediction of the likelihood of a splice site being functional (positive values) or nonfunctional (negative values), reaching from -100% to +100%. ΔScore (DeltaScore): the difference between the score of the splice site on the reference sequence and the score of the splice site on the variant sequence.

Table S4: Results of bioinformatic tools for novel mutations in *ALOXE3*

**Mutation**		**gnomAD**	**rs number**	**ClinVar**	**MutationTaster**	**Polyphen-2 (HumVar)**	**Provean (cutoff=-2.5)**	**SIFT (cutoff=0.05)**	**fathmm**	**NNSplice**	**NetGene2**	**SSP (VarSEAK)**
**c.57_63del**	**p.(Asp20Serfs*17)**	n/a	n/a	n/a	disease causing (1.0)	-	-	-	-	-	-	-
**c.271G>T**	**p.(Glu91*)**	0x homo, 2x het, MAF 0.0007%	rs367827093	n/a	disease causing (1.0)	-	-	-	-	-	-	-
**c.308A>C**	**p.(Gln103Pro)**	n/a	n/a	n/a	disease causing (0.999)	probably damaging (0.986)	deleterious (-4.59)	damaging (0.008)	tolerated (-0.17)	-	-	-
**c.397A>G**	**p.(Arg133Gly)**	0x homo, 9x het, MAF 0.0036%	rs373520842	n/a	disease causing (0.999)	probably damaging (0.992)	deleterious (-6.03)	damaging (0.001)	damaging (-2.50)	-	-	-
**c.680+1G>A**	**p.?**	0x homo, 1x het, MAF 0.0004%	rs764781178	1x pathogenic	-	-	-	-	-	Loss of donor splice site	Loss of donor splice site	No GT, loss of function for authentic splice site
**c.758del**	**p.(Phe253Serfs*27)**	n/a	n/a	n/a	disease causing (1.0)	-	-	-	-	-	-	-
**c.833A>C**	**p.(Tyr278Ser)**	n/a	n/a	n/a	disease causing (0.997)	probably damaging (0.930)	deleterious (-6.63)	damaging (0.000)	tolerated (-0.98)	-	-	-
**c.923T>C**	**p.(Leu308Pro)**	0x homo, 4x het, MAF 0.0016%	rs764052154	n/a	disease causing (0.999)	probably damaging (1.000)	deleterious (-6.21)	damaging (0.002)	damaging (-1.83)	-	-	-
**c.952dup**	**p.(Leu318Profs*58)**	0x homo, 1x het, MAF 0.0032%	rs147208742 1	n/a	disease causing (1.0)	-	-	-	-	-	-	-
**c.957G>A**	**p.(Glu319=)**	0x homo, 1x het, MAF 0.0004%	rs142777070 3	n/a	disease causing (1.0)	-	neutral (0.00)	tolerated (1.000)	-	Loss of authentic donor splice site, use of a cryptic site 4 nt downstream	Reduced score for donor splice site (wt 0.95 → mut 0.70). Cryptic splice site 4 nt downstream (0.95)	Strong decrease of score for authentic splice site (Δ -61.89%). Use of a cryptic site 4 nt downstream
**c.1031A>C**	**p.(Gln344Pro)**	0x homo, 13x het, MAF 0.0052%	rs142781546	n/a	disease causing (1.0)	probably damaging (0.999)	deleterious (-5.33)	damaging (0.002)	damaging (-2.73)	-	-	-
**c.1061G>A**	**p.(Trp354*)**	n/a	rs767046669	n/a	disease causing (1.0)	-	-	-	-	-	-	-
**c.1164G>T**	**p.(Trp388Cys)**	n/a	n/a	n/a	disease causing (1.0)	probably damaging (1.000)	deleterious (-12.4)	damaging (0.000)	damaging (-5.73)	-	-	-

**c.1193C>T**	**p.(Ser398Phe)**	n/a	n/a	n/a	disease causing (0.650)	possibly damaging (0.760)	deleterious (-3.88)	damaging (0.000)	tolerated (-1.19)	-	-	-
**c.1202T>A**	**p.(Leu401Gln)**	0x homo, 1x het. MAF 0.0004%	rs761241768	n/a	disease causing (0.732)	benign (0.261)	neutral (-0.40)	tolerated (0.313)	tolerated (-0.94)	-	-	-
**c.1246T>C**	**p.(Cys416Arg)**	n/a	n/a	n/a	disease causing (1.0)	benign (0.012)	neutral (-1.99)	damaging (0.021)	tolerated (-0.98)	-	-	-
**c.1292dup**	**p.(His431Glnfs*90)**	0x homo, 1x het, MAF 0.0004%	rs156799943 1	n/a	disease causing (1.0)	-	-	-	-	-	-	-
**c.1393-1G>A**	**p.?**	n/a	n/a	n/a	-	-	-	-	-	Loss of acceptor splice site	Loss of acceptor splice site	No AG, loss of function for authentic splice site
**c.1786-2A>G**	**p.?**	0x homo, 4x het, MAF 0.0016%	rs139375856	1x pathogenic	-	-	-	-	-	-	-	No AG, loss of function for authentic splice site
**c.1786-63_1807del**	**p.?**	n/a	n/a	n/a	-	-	-	-	-	-	-	-
**c.1804dup**	**p.(Met602Asnfs*3 0)**	n/a	n/a	n/a	disease causing (1.0)	-	-	-	-	-	-	-
**c.1812T>A**	**p.(Asn604Lys)**	n/a	n/a	n/a	disease causing (0.996)	probably damaging (0.997)	deleterious (-5.78)	damaging (0.001)	damaging (-1.93)	-	-	-
**c.1937_1944del**	**p.(Ser646Thrfs*13)**	n/a	n/a	n/a	disease causing (1.0)	-	-	-	-	-	-	-
**c.1954C>T**	**p.(Gln652*)**	0x homo, 1x het, MAF 0.0004%	rs116328086 6	1x not provided	disease causing (1.0)	-	-	-	-	-	-	-
**deletion exon 15**	**p.? gross deletion**	-	-	-	-	-	-	-	-	-	-	-

n/a: not listed in the database. homo: homozygous. het: heterozygous. MAF: minor allele frequency. -: not evaluated. nt: nucleotides. wt: wildtype, mut: mutation. Scores are in brackets.
gnomAD: The data set provided on this website spans 125,748 exome sequences and 15,708 whole-genome sequences from unrelated individuals sequenced as part of various disease-specific and population genetic studies.
Mutation Taster: The probability value is the probability of the prediction. A value close to 1 indicates a high security of the prediction.
PolyPhen-2: HumVar prediction, the score ranges from 1.0 (damaging) to 0.0 (benign).
Provean: Cutoff threshold is -2.5. Variants with a score equal to or below -2.5 are considered "deleterious", variants with a score above -2.5 are considered "neutral".
SIFT: Cutoff threshold is 0.05. Substitutions with less than 0.05 are predicted as damaging.
Fathmm: Prediction algorithm: weighted. Scores approximately equal to zero indicate no significant change in the underlying amino acid probabilities, scores less than zero indicate an unfavorable substitution, scores greater than zero indicate a favorable substitution.
NNSplice: Splice site predictions for 1 sequence with donor score cutoff 0.40, acceptor score cutoff 0.40. Score ranging from 0 to 1, higher score implies a more potential splice site.
NetGene2: Cutoff values used for confidence: highly confident donor sites (H): 95.0 %, nearly all true donor sites: 50.0 %, highly confident acceptor sites (H): 95.0 %, nearly all true acceptor sites: 20.0 %. Confidence score ranging from 0 to 1, higher score implies a higher confidence of true site.
SSP (VarSEAK): Score: Prediction of the likelihood of a splice site being functional (positive values) or nonfunctional (negative values), reaching from -100% to +100%. ΔScore (DeltaScore): the difference between the score of the splice site on the reference sequence and the score of the splice site on the variant sequence.

Table S5: Distribution of mutations in *ALOX12B* and *ALOXE3*

ALOX12B:

Exon	Amino acids	Number of amino acids	Number of missense mutations	Number of all mutations	Number of amino acids divided by the number of missense mutations	Number of amino acids divided by the number of all mutations	Number of missense variants in gnomAD	Number of amino acids divided by the number of missense variants in gnomAD
1	1-49	49	6	8	8.2	6.13	19	2.58
2	50-117	68	8	13	8.5	5.23	52	1.31
3	118-144	27	3	6	9	4.5	11	2.45
4	145-175	31	1	4	31	7.75	24	1.29
5	176-216	41	1	4	41	10.25	12	3.41
6	217-251	35	1	2	35	11.67	13	2.69
7	252-309	58	6	9	9.67	6.44	36	1.61
8	310-357	48	7	16	6.86	3	19	2.53
9	358-425	68	16	20	4.25	3.4	27	2.52
10	426-454	29	8	11	3.63	2.64	19	1.53
11	455-510	56	12	14	4.67	4	32	1.75
12	511-551	41	9	13	4.56	3.15	24	1.71
13	552-585	34	7	12	4.86	2.83	9	3.78
14	586-642	57	9	11	6.33	5.18	25	2.28
15	643-701	59	9	15	6.5	3.69	29	2.03

ALOXE3:

Exon	Amino acids	Number of amino acids	Number of missense mutations	Number of all mutations	Number of amino acids divided by the number of missense mutations	Number of amino acids divided by the number of all mutations	Number of missense variants in gnomAD	Number of amino acids divided by the number of missense variants in gnomAD
2	1-49	49	0	2	-	24.5	19	2.58
3	50-117	68	1	5	68	13.6	48	1.42
4	118-144	27	1	3	27	9	17	1.59
5	145-184	40	0	0	-	-	25	1.6
6	185-226	42	0	2	-	21	37	1.14
7	227-261	35	0	3	-	11.67	19	1.84
8	262-319	58	4	7	14.5	8.29	31	1.87
9	320-367	48	1	3	48	16	35	1.37
10	368-435	68	7	11	9.71	6.18	32	2.13
11	436-464	29	1	2	29	14.5	11	2.64
12	465-520	56	1	2	56	28	45	1.24
13	521-561	41	0	1	-	41	27	1.52
14	562-595	34	0	0	-	-	23	1.48
15	596-652	57	2	6	28.5	9.5	29	1.97
16	653-711	59	0	2	-	29.5	38	1.55

Amino acids beginning at the end of an exon were assigned to the next exon. Large deletions, in frame variants and start loss variants were not included.
